# Supplementary material for: Effect of new urbanization on cities’ innovation in China: Evidence from a quasi-natural experiment of a comprehensive pilot
Source: PLoS One. 2023 Apr 21;18(4):e0284772. doi: 10.1371/journal.pone.0284772 (PMC10121039; doi:10.1371/journal.pone.0284772)
Supplement: Attachment — Submitted filename: Response to Reviewers 2022.11.07.docx [file pone.0284772.s001.docx]

Dear Dr. Abdul Majeed and Reviewers,

Thank you for your and the reviewers’ comments on our manuscript PONE-D-22-18878 titled *Effect of new urbanization on cities' innovation in China: Evidence from a quasi-natural experiment of a comprehensive pilot.* Those comments were valuable and very helpful for revising and improving our paper besides providing significant guidance for our research. We have carefully studied the comments and made revisions in the hope of the final approval. The main revisions in the paper and our responses to the reviewer’s comments are as follows:

**Response to the reviewer’s comments: Reviewer: 1**

**Comments to the Author:**

1. In section literature review, you mentioned this sentence "The second is the impact of urbanization on urban innovation". This is consistent with the content of the previous sentence "The first is the impact of urbanization on urban innovation". Do you want to express the second is the impact mechanism of urbanization on urban innovation?

2. It is necessary to supplement the relevant literature on talent agglomeration, industrial structure, and income scale.

3. It is necessary to provide descriptive statistics to get a general idea of the data.

4. In section placebo test, the regression coefficient should be insignificant to exclude other policy interference. However, the result of this paper is significantly negative. Of course, this is related to changing the treatment group and the control group. The common operation is to advance the quasi-natural experiment time by several years or randomly select the quasi-natural experiment time. This paper can consider a similar method.

5. Is it reasonable that industrial structure is both a control variable and an intermediary variable? Although one uses the output value, the other uses the added value.

6. Please check the grammar and format (such as references [14] and [18]).

**Response:** Thanks a lot for your encouragement. We greatly appreciate your time and very professional and helpful comments. Our responses are provided as follows.

**Point1:** In section literature review, you mentioned this sentence "The second is the impact of urbanization on urban innovation". This is consistent with the content of the previous sentence "The first is the impact of urbanization on urban innovation". Do you want to express the second is the impact mechanism of urbanization on urban innovation?

**1. Answer:** Following the reviewer’s suggestions, We have rewritten the literature review.

**Literature review**

The initial research regarding the impact of urbanization on technological innovation primarily focused on the effects of urbanization development on invention patents. Researchers found that cities are more conducive to generating invention patents than rural areas. Pred (1966) examined the 50-year experience data of 35 major cities in the United States from 1860 to 1910 as a sample; the findings indicate that the number of patents per capita in major cities in the United States was far higher than the national average [9]. Higgs (1971) examined this same data and concluded that there was a significant relationship between urbanization level and the number of patent applications in 1870-1920, with a positive correlation [10]. To prove the universality of the impact of urbanization on invention patents, Laumas and Williams (1984) expanded the study beyond the United States by including industrialized countries such as Britain, France, and Germany as the research subjects. They verified the relationship between city size and technological innovation, and concluded that city size is proportional to the number of inventions [11]. Based on these predecessors, experts further investigated the impact of urbanization on invention patents. For example, Jaffe et al. (1993) and Feldman et al. (1999) found that patent inventions have significant imitation characteristics in the urbanization process and that having various innovation subjects in a city can improve innovation capacity through shared encouragement [12-13].

Regarding the fundamental mechanism of urbanization in promoting technological innovation, the basic theory of urban economics holds that urbanization promotes the improvement of technological innovation through a spatial economic structure and knowledge diffusion. Cities are important places for innovation elements and knowledge spillovers. Researchers have examined the impact of spatial distance on knowledge diffusion and technological innovation. For example, Krugman (1991) and Feldman (1994) emphasized that spatial distance is one of the main obstacles to knowledge spillover, while zero-distance communication may have a significant impact on the diffusion of knowledge, technology, and other innovative elements; hence, aggregation can significantly impact technological innovation [14-15]. Regarding the effects of urbanization on knowledge diffusion and technological innovation, Black and Henderson (1999) suggest that knowledge spillovers lead to agglomeration, and the urbanization process can promote knowledge spillovers [16]. Rosenthal and Strange (2004) and Tappeiner (2008) pointed out that urban scale expansion and spatial agglomeration have formed a dynamic environment, which is conducive to the interaction and information exchange between people; this produces the externality of knowledge and fosters innovation [17-18]. Wang and Ren (2022) also found that urbanization development optimizes environmental, scientific, and technological innovation; this innovation enables the gathering and shortening of the space, time, and cost distance between them [19]. Regarding the impact of urbanization on the diffusion and application of new technologies, Zhang and Huang (2022) found that urbanization can accelerate the diffusion of scientific and technological innovation among large, small, and medium-sized cities and boost the application of new technologies [20]. Therefore, urbanization can promote the production of urban technological innovation and provide an effective diffusion channel for existing technological innovation.

In China, the role of urbanization in technological innovation has inspired interest in the academic community and has been studied from different perspectives. Some research has focused on the impact of urbanization on urban innovation. Cheng (2010) found that urbanization created a suitable environment, accelerated knowledge spillover and innovation diffusion, and thus promoted urban innovation and development [21]. Qiu and Li (2017) examined urban agglomeration in the middle reaches of the Yangtze River in China to analyze the role of urbanization in promoting urban innovation [22]. Liu et al. (2017) focused on innovation efficiency. Their study found that the impact of urbanization on technological innovation efficiency comprises a U-shaped, nonlinear relationship; when the urbanization level crosses a particular critical value, it will promote the improvement of technological innovation efficiency [23]. Another focus of research has been the interaction between urbanization and urban innovation. Cheng and Li (2008) found that China's urbanization and technological innovation exhibit a Granger causality but that urbanization plays a greater role in urban technological innovation. In contrast, urban technological innovation plays a weaker role in urbanization [24]. Zhang et al. (2017) studied the urban agglomeration in China's middle reaches of the Yangtze River. They found a time lag in the dynamic relationship between urbanization and technological innovation. The impact between the two changes from inhibition to promotion over time; further, the impact of urbanization on technological innovation should be stronger than that of technological innovation on urbanization [25]. Ma et al. (2020) studied the Beijing Tianjin Hebei urban agglomeration in China and concluded that the interaction between urbanization and technological innovation is significant. However, they found a large spatial difference in the coupling relationship [26].

Scholars at home and abroad have conducted extensive research on the relationship between urbanization and urban innovation and have made many valuable research achievements. However, there is room for further exploration and discussion based on the existing literature. First, few studies evaluate the impact of China's new urbanization pilot policy on urban innovation. Second, in terms of research methods, most existing studies adopt ordinary panel regression or spatial econometric models. Few researchers regard the comprehensive pilot project of China's new urbanization as a quasi-natural experiment and use the multi-period double difference model (DID) to investigate the policy effect of pilot policies on urban innovation.

Can the pilot policy of new urbanization boost urban innovation? Further data analysis and empirical tests are urgently needed to investigate this research question. Based on the gap in the literature, this paper examines cities at the prefecture level and above from 2008 to 2019 as the research samples. We regard the new urbanization pilot as a quasi-natural experiment and use the multi-period DID model to investigate the policy effect of new urbanization pilot on urban innovation. We provide policy references for comprehensively promoting new urbanization and improving urban innovation capacity. The originality of this paper is as follows: (1) adopting China's new urbanization pilot policy as the research theme; (2) the multi-period DID model is used to analyze the policy effect of new urbanization pilot on urban innovation; (3) our research model tests the intermediary effect of talent gathering, industry gathering, and income scale on urban innovation.

**Point2:** It is necessary to supplement the relevant literature on talent agglomeration, industrial structure, and income scale.

**2. Answer:** Thank you for your valuable questions and professional suggestions. We have supplemented relevant literature. Which is shown as below.

**Agglomeration of human capital**

In the urbanization process, many factors at the city level, such as wages, housing prices, employment opportunities, and public services, can impact labor mobility [27]. Generally, the larger a city, the higher the level of its human capital [28]. For example, Bertinelli and Black (2004) studied the relationship between urbanization and human capital promotion by taking 30 years' time series economic data of 100 countries as samples; they found that human capital would increase by 0.144 units on average for every percentage point increase in urbanization, an increase of 0.72 years of schooling on average [29]. China's new urbanization emphasizes "people-oriented" capital. "Population urbanization" is fundamental to quickening the development of high-quality urbanization and promoting the overall development of social harmony and progress. Population gathering provides the advantage of "location proximity" for disseminating and exchanging knowledge, technology, and data [30]. The pilot policy of China's new urbanization identifies specific plans for upgrading vocational skills, including employment skills training, post-skills training, highly skilled talents, and entrepreneurship training, while providing human capital for innovation. The classic endogenous economic growth theory holds that human capital is at the core of technological progress and economic growth; human capital is the core element of innovation output [31]. Therefore, as cities expand, the per capita education level is enhanced, and communication will become more convenient and frequent, which is crucial to improving a city's innovation capacity [32].

**Promoting industrial agglomeration**

Urbanization can provide conditions for industrial economic growth by creating a suitable development environment [33]. Moreover, urbanization and industrial structure upgrades are interrelated; there is a long-term, balanced relationship between urbanization, industrialization, and the development of the tertiary industry [34]. Second, the size and quality of the labor force significantly improves during the urbanization process. High-quality human capital has promoted industrial agglomeration. Urbanization is a process of talent flow, innovative technological production, industrial upgrading. China's new urbanization will change the extensive development mode of traditional urbanization. The key lies in transforming the mode of production, improving the utilization rate of resources, and upgrading and promoting the agglomeration of industrial structures. In the process of industrial agglomeration, many enterprises perform concentrated economic activities in cities, which can lead to local knowledge- and infrastructure-sharing, spillovers, and a larger labor market. This provides the necessary conditions to generate innovation. Therefore, under specific inputs and technology levels, urbanization increases economic efficiency through the industrial agglomeration effect, and promotes a spillover effect to accelerate the diffusion of technological innovation and improve innovation efficiency [35].

**Expanding income scale**

Regarding the impact of urbanization on income, Lewis (1954) put forward the famous "dual economic structure" model, claiming that income inequality distribution will continue to increase during the initial stages of economic development. As urbanization accelerates, the expansion of the urban economy and overall income level will increase, and the shortage of surplus labor will lead to a gradual decline in income inequality [36]. Similarly, the Todaro model (Todaro, 1969) posits that the labor force will gradually transition from rural areas to cities and towns due to the expected income gap between urban and rural areas. The transfer of labor will improve the degree of equalization of labor remuneration, thereby improving the income level of rural residents. Income distribution and its evolution influence the innovation mechanism through demand and subsequently affect the allocation of innovation resources [37]. On the one hand, the widening income gap enables monopoly manufacturers to charge higher product prices from high-income groups, and the price effect strengthens the allocation of innovation resources. On the other hand, income is more concentrated, reducing the proportion of people with purchasing power; then, the market scale effect inhibits R&D and innovation activities [38]. From a dynamic perspective, the revenue growth rate reflects the expected changes in market scale expansion, which accelerates the entry of innovative manufacturers [9] and reduces the uncertainty of expected profits and operational risks, further encouraging R&D innovation [40].

**Point3:** It is necessary to provide descriptive statistics to get a general idea of the data.

**3. Answer:** Thank you for your professional advice. We have supplemented according to the comments of the reviewers.

**Table 3** Descriptive Statistics

| **Symbol** | **Mean value** | **Standard deviation** | **Minimum value** | **Maximum**  **value** | **Sample size** |
| --- | --- | --- | --- | --- | --- |
| *UIC* | 0.0937 | 0.0994 | 0.0057 | 0.7603 | 2316 |
| *did* | 0.1913 | 0.3934 | 0 | 1 | 2316 |
| *indu* | 0.4800 | 0.1003 | 0.1474 | 0.8508 | 2316 |
| *pop* | 5.8708 | 0.6389 | 3.7842 | 7.3132 | 2316 |
| *hum* | 0.0735 | 0.0658 | 0.0031 | 0.5559 | 2316 |
| *fin* | 0.9237 | 0.6068 | 0.1122 | 7.4502 | 2316 |

**Point4:** In section placebo test, the regression coefficient should be insignificant to exclude other policy interference. However, the result of this paper is significantly negative. Of course, this is related to changing the treatment group and the control group. The common operation is to advance the quasi-natural experiment time by several years or randomly select the quasi-natural experiment time. This paper can consider a similar method.

**4. Answer:** Thank you for your professional suggestions. Following the reviewer’s suggestions, we have further discussed the placebo test.. Which is shown as follows.

**Placebo test**

*Sample replacement*. Before the new urbanization policy was implemented, there was no noticeable difference in the innovation capacity of each city. As such, we performed a placebo test on the samples using an alternative method. The specific approach of this method is to replace the treatment group samples with the control group samples, replace the control group samples with the treatment group samples, and then conduct the DID estimation. The placebo test results in column (4) of Table 5 show that the estimation coefficient is significantly negative at 1%. This indicates that the new urbanization policy had no positive impact on the innovation capacity of pilot cities excluded from the policy, which further confirms the reliability of the DID estimation results in this study.

*Change in time*. To further confirm the effect of the new urbanization policy, the implementation time of the policy was advanced three years as a virtual policy time point. We determined this as the placebo test of the policy effect [47]. If the test results indicate no similar causal relationship, i.e., if the virtual policy cannot make significant changes in urban innovation, it confirms that the new urbanization policy indeed causes the changes in urban innovation estimated by the double difference above. The regression coefficients of the dummy variables of the pilot policy in column (5) of Table 5 are not significant. This indicates that before the promulgation of the new urbanization policy, there is no significant difference between the pilot and non-pilot cities regarding technological innovation. This finding further confirms the impact of the new urbanization policy on urban innovation.

**Table 5** Placebo test

| Explanatory variable | (1) | (2) 2014 | (3) 2015 | (4) | (5) |
| --- | --- | --- | --- | --- | --- |
| *did* | 0.0133***  （0.0042） | 0.0169**  （0.0073） | 0.0162**  （0.0067） | -0.0155***  （0.0055） | 0.0031（0.0034） |
| Constant | -0.3100*  （0.1738） | -0.3498*  （0.1854） | -0.3787*  （0.1929） | -0.3399*  （0.1756） | -0.3766**（0.1790） |
| Sample size | 1073 | 2016 | 2100 | 2316 | 2316 |
| R^2^ | 0.2940 | 0.2175 | 0.2236 | 0.2254 | 0.2078 |

Note: Each regression incorporates the control variables, time fixed effects, and individual fixed effects; results are not shown.

**Point5:** Is it reasonable that industrial structure is both a control variable and an intermediary variable? Although one uses the output value, the other uses the added value.

**5. Answer:** Thanks a lot for your very professional and helpful comments. The author appreciate the reviewers for their valuable comments about the intermediary variable between the new town urbanization policy and urban innovation of our article.Following the reviewer’s suggestions, we have replaced the industrial structure with industrial agglomeration. Which is shown as follows.

**Intermediary effect analysis**

To evaluate the action mechanism of the new urbanization pilot policy on urban innovation capacity, this study draws on the analysis of Baron and Kenny (1986) and other intermediary effect models for reference to construct the intermediary effect model in formulas (3) to (5) [48]:

$UIC_{it}=\alpha_{0}+\alpha_{1}did_{it}+\sum\delta_{k}year_{k}+\mu_{city}+\varepsilon_{it}$ (3)

$M_{it}=\lambda_{0}+\lambda_{1}did_{it}+\sum\delta_{k}year_{k}+\mu_{city}+\varepsilon_{it}$ (4)

$UIC_{it}=\varphi_{0}+\varphi_{1}did_{it}+\varphi_{2}M_{it}+\sum\delta_{k}year_{k}+\mu_{city}+\varepsilon_{it}$ (5)

Where *M* represents an intermediary variable.

Based on the availability of data, this paper analyzes the effect of talent gathering, income scale effect, and industrial gathering effect. The definition of other variables is the same as that of equation (1). We measured talent agglomeration effect (talent) by the proportion of the non-agricultural employment population of the total urban population. The location quotients measure the industrial agglomeration effect (lq) [49]. Based on the existing practice [50], the specific expression is as follows:

$lq_{it}=(M_{it}/M_{t})/(P_{it}/P_{t})$ （6）

In formula (6), *lq_it_* represents the location entropy of the secondary industry of city *i* in year *t*. The higher the value, the higher the industry concentration degree in the region. *M_it_* is the number of secondary industry employees in the city in year *t*; *M_t_* is the number of secondary industry employees in the country in year *t*; *P_it_* is the number of urban employees in year *t*; *P_t_* is the number of employees in year *t*; The income scale effect (pgdp) is measured by per capita GDP.

The mediating effect models of formulas (3) to (5) were regressed, and the significance of the action mechanism of mediating variables was tested based on the bootstrap method. Columns (1) and (2) of Table 7 portray the regression results of the pilot policies on talent agglomeration and the regression results of pilot policies and talent agglomeration on urban innovation, respectively. In column (1), the regression coefficient of the new urbanization pilot policy on talent agglomeration is positive and significant at 1%. This indicates that the pilot policy is conducive to urban talent agglomeration. In column (2), the regression result coefficient of talent gathering on urban innovation is significant at 1% and is positive. This indicates that talent gathering can promote the improvement of urban innovation capacity. Columns (1) and (2) jointly illustrate that the new urbanization pilot can effectively promote talent agglomeration, provide a good foundation for urban innovation, and improve urban innovation capacity. The intermediary effect coefficient of talent gathering is 0.0167, accounting for 25.57%. The results of the Sobel and Bootstrap tests are significant at 1%, which verifies the effectiveness of the intermediary effect of talent gathering.

Columns (3) and (4) report the test results of new urbanization pilot policies on industrial agglomeration and the regression results of pilot policies and industrial agglomeration on urban innovation, respectively. Column (3) indicates that the regression result coefficient of the new urbanization pilot policy on industrial agglomeration is significant at 1% and is positive. This suggests that the new urbanization pilot can promote industrial agglomeration. In column (4), the regression result coefficient of industrial agglomeration to urban innovation is also significant at 1% and was positive. This indicates that changes in industrial agglomeration can improve urban innovation capacity. Combined with the results of columns (3) and (4), our results show that the pilot project of new urbanization can promote industrial agglomeration and thus positively impact urban innovation capacity. The intermediary effect coefficient of industrial agglomeration is 0.0091, accounting for 13.97%. The results of the Sobel and Bootstrap tests support the effectiveness of the intermediary effect of industrial agglomeration.

Column (5) reports the regression analysis results of the pilot policy on income scale. Column (6) provides the regression analysis results of the pilot policy and income scale on urban innovation. In column (5), the regression analysis coefficient of the pilot policy on income scale is significantly positive at 1%. This indicates that the new urbanization pilot helps improve income level. In column (6), the regression coefficient of the income scale to urban innovation is significant at 1% and is positive. This indicates that income growth can significantly promote urban innovation. Columns (5) and (6) jointly show that the pilot policy will increase income and help improve urban innovation capacity. The intermediary effect coefficient of the income scale is 0.0252, accounting for 38.6%. The Sobel and Bootstrap tests results are significant at a high level, confirming the intermediary effect of the income scale.

**Table 7** Intermediary effect test.

| Variable | *talent* (1) | *UIC* (2) | lq (3) | *UIC* (4) | *Pgdp* (5) | *UIC* (6) |
| --- | --- | --- | --- | --- | --- | --- |
| *did* | 0.0351***  （0.0058） | 0.0485***  （0.0043） | 0.0931***  （0.0140） | 0.0561***  （0.0049） | 0.2503***  （0.0285） | 0.0400***  （0.0030） |
| *talent* |  | 0.4742***  （0.0152） |  |  |  |  |
| lq |  |  |  | 0.0978***  （0.0073） |  |  |
| *pgdp* |  |  |  |  |  | 0.1005***  （0.0031） |
| Constant | 0.0818***（0.0243） | -0.3081***  （0.0178） | -0.1961***  （0.0586） | -0.2502***  （0.0204） | 9.0512***  （0.1191） | -1.1790***  （0.0327） |
| Sobel test | 0.0167  （z=5.927，p=0.000） | | 0.0091  （z=5.955，p=0.000） | | 0.0252  （z=8.484，p=0.000） | |
| Bootstrap test (Indirect effect) | 0.0166  （z=4.94，p=0.000） | | 0.0119  （z=7.60，p=0.000） | | 0.0328  （z=10.81，p=0.000） | |
| Bootstrap test (  direct effect) | 0.0337  （z=7.83，p=0.000） | | 0.0384  （z=9.64，p=0.000) | | 0.0175  （z=3.31，p=0.000） | |
| Proportion of intermediary effect (%) | 25.57 | | 13.97 | | 38.60 | |
| Sample size | 2316 | | 2316 | | 2316 | |
| R^2^ | 0.2380 | 0.5289 | 0.3376 | 0.3791 | 0.5505 | 0.5542 |

**Point 6:** Please check the grammar and format (such as references [14] and [18]).

**6.** **Answer:** Thanks a lot for your encouragement, as well as your time and very professional and helpful comments. This kind suggestion is well taken. We have tried our best to carefully check the expressions. Moreover, the revised manuscript has been professionally proofread by a native English speaker.

We would like to extend our sincere gratitude for these helpful comments.

**Response to the reviewer’s comments:  Reviewer: 2**

**Comments to the Author:**

This paper mainly analyzes and evaluates the effect of the comprehensive pilot policy of new urbanization on cities' innovation in China, and regards the national comprehensive pilot of new urbanization as a quasi natural experiment and adopts multi-period difference-in-difference (DID) models to investigate the policy effect. This work is interesting and useful for further studies. However, this paper needs to minor revise before publication.

1. Abstract needs to rewrite. The current version of abstract dose not provide the necessary information surrounding the method, findings, results, and policy recommendations, etc.

2. Clearly demonstrating the key objective of this work in the Introduction is useful. Additionally, literature review needs to be further improved.

3. This study does not theoretically analyze the nexus between the public policy and cities' innovation; therefore, the author should add some contents to improve the theoretical analysis in the revised process.

4. Policy recommendations in this study are also weak, please improve the contents.

5. Adding limitations and further research directions is also useful.

6. Language quality of the paper should be improved.

**Response:** Thanks a lot for your encouragement. We greatly appreciate your time and very professional and helpful comments. Our responses are provided as follows.

**Point1:** Abstract needs to rewrite. The current version of abstract dose not provide the necessary information surrounding the method, findings, results, and policy recommendations, etc.

**1. Answer:** Following the reviewer’s recommendations, we have added some quantities to the abstract. The abstract is shown as below:

Urbanization drives the concentration of innovation elements and knowledge diffusion, which is conducive to enhancing regional innovation capacity. To meet the developmental needs of the new era, China has released the National New Urbanization Plan (2014-2020) to test the effect of this policy on urban innovation. The 2014 comprehensive pilot project of China's new urbanization strategy is considered a quasi-natural experiment. This study examines relevant data from cities at the prefecture level and above from 2008 to 2019 using DID, PSM-DID, intermediary effect, and other methods. We established an empirical model to investigate the impact of new urbanization pilot policies on urban innovation. The results indicate that the new urbanization pilot policy can significantly promote an increase in urban innovation capacity. The heterogeneity of the impact of new urbanization pilot policies is evident in the improvements to urban grade, region, scale, etc. Regarding function mechanism, new urbanization can promote urban innovation through talent gathering, industrial gathering, income scale, and other effects. This paper posits the importance of summarizing the success of the pilot project of new urbanization and explores new urbanization to promote urban innovation from multiple perspectives. We put forth recommendations based on the experience of different cities to implement an urban innovation differentiation strategy in constructing new urbanization.

**Point2:** Clearly demonstrating the key objective of this work in the Introduction is useful. Additionally, literature review needs to be further improved.

**2. Answer:** Thank you for your professional suggestions. According to the reviewer’s comments, we have improved the Introduction and literature review. Which is shown as below.

**Introduction**

Urbanization is accompanied by industrialization, the rise of non-agricultural industry, and the migration of rural populations to cities and towns. Since the Industrial Revolution, urbanization has become one of the leading factors in economic development, there is a high correlation between urbanization and economic development. Zhen (2013) found that the average logarithmic correlation coefficient between the urbanization rate and GDP per capita of major developed countries worldwide was 0.85, which confirms the relationship between urbanization level and economic development. In contrast, the correlation coefficient between 1978 and 2014 was 0.99, indicating that China's economic development and urbanization rates are more prominent [1]. Urbanization has increased China's potential for economic development and has become an essential factor in boosting China's economic growth. Since 1978, China's urbanization has developed rapidly with the gradual breakdown of the urban-rural "dual structure" and the liberalization of population mobility control. By the end of 2021, China's urban population accounted for 64.72% of the total population.

China's traditional urbanization path has been dominated by extensive developments in scale, quantity, and speed [2]. Increased urbanization has led to the rampant expansion of medium- and large-sized cities, degradation of water and soil resources, ecological damage, and other problems, which are becoming increasingly common [3]. Furthermore, this traditional urbanization model, i.e., increasing population proportion and urban area expansion, has also caused economic development problems. For example, slow optimization and adjustment of the industrial structure and the uncoordinated development of industrialization and urbanization have led to a widening income gap between urban and rural residents [4]. The "synergy" between industrial structure and urbanization is not apparent, and the "structural slowdown" of economic development in China has been significant [5]. The urban economy relies too heavily on factor-driven and investment-driven development. It pays insufficient attention to improving urban scientific and technological innovation capacity, leading to the inadequate innovation-driven development capacity of cities [6].

However, China's economy has entered a new stage of high-quality development. The economic development mode oriented by quantitative growth urbanization will not be sustainable during this new stage. Various problems that have accumulated during the process of traditional urbanization need to be solved by changing the direction of urbanization to adjust the mode of economic development. Therefore, the new urbanization policy, which differs from traditional urbanization, is considered the most effective way to achieve high-quality urban economic development [7].

Urbanization is characterized by the influx of labor, capital, land, and other elements to cities and towns; this leads to positive technology spillover and diffusion effects conducive to promoting urban technological innovation [8]. Promoting new urbanization has become an important strategic goal in China's development plans. To grasp the opportunities provided by urbanization, researchers and policymakers need to examine the new characteristics and trends of urbanization development, explore and optimize the development mode of urbanization, and encourage the innovative vitality of urban and rural economies. In 2014, China released the National Plan for Innovative Urbanization (2014-2020) (hereafter referred to as the Plan). The Plan provides strategic guidance and task scheduling for new urbanization and specifies that policy pilots will be conducted in batches in multiple regions across the country. The Plan also proposes that "new urbanization should conform to the new trend of scientific and technological progress and industrial reform, give play to the role of urban innovation carrier, rely on the advantages of science and technology, education and human resources, and promote the city to take the path of innovation-driven development," which provides a viable path to promote urban innovation using the new urbanization policy.

However, can implementing the pilot policy of new urbanization promote urban innovation? Does this influence have different heterogeneity? How does this influence arise? This study examines the new urbanization pilot policy as a quasi-natural experiment to address these research questions. We use DID, PSM-DID, intermediary effect, and other methods to analyze the policy impact of new urbanization on urban innovation. Our findings indicate a positive impact on improving our urban innovation system, accelerating the development of innovative cities, and promoting the rational formulation of new urbanization policies.

**Literature review**

The initial research regarding the impact of urbanization on technological innovation primarily focused on the effects of urbanization development on invention patents. Researchers found that cities are more conducive to generating invention patents than rural areas. Pred (1966) examined the 50-year experience data of 35 major cities in the United States from 1860 to 1910 as a sample; the findings indicate that the number of patents per capita in major cities in the United States was far higher than the national average [9]. Higgs (1971) examined this same data and concluded that there was a significant relationship between urbanization level and the number of patent applications in 1870-1920, with a positive correlation [10]. To prove the universality of the impact of urbanization on invention patents, Laumas and Williams (1984) expanded the study beyond the United States by including industrialized countries such as Britain, France, and Germany as the research subjects. They verified the relationship between city size and technological innovation, and concluded that city size is proportional to the number of inventions [11]. Based on these predecessors, experts further investigated the impact of urbanization on invention patents. For example, Jaffe et al. (1993) and Feldman et al. (1999) found that patent inventions have significant imitation characteristics in the urbanization process and that having various innovation subjects in a city can improve innovation capacity through shared encouragement [12-13].

Regarding the fundamental mechanism of urbanization in promoting technological innovation, the basic theory of urban economics holds that urbanization promotes the improvement of technological innovation through a spatial economic structure and knowledge diffusion. Cities are important places for innovation elements and knowledge spillovers. Researchers have examined the impact of spatial distance on knowledge diffusion and technological innovation. For example, Krugman (1991) and Feldman (1994) emphasized that spatial distance is one of the main obstacles to knowledge spillover, while zero-distance communication may have a significant impact on the diffusion of knowledge, technology, and other innovative elements; hence, aggregation can significantly impact technological innovation [14-15]. Regarding the effects of urbanization on knowledge diffusion and technological innovation, Black and Henderson (1999) suggest that knowledge spillovers lead to agglomeration, and the urbanization process can promote knowledge spillovers [16]. Rosenthal and Strange (2004) and Tappeiner (2008) pointed out that urban scale expansion and spatial agglomeration have formed a dynamic environment, which is conducive to the interaction and information exchange between people; this produces the externality of knowledge and fosters innovation [17-18]. Wang and Ren (2022) also found that urbanization development optimizes environmental, scientific, and technological innovation; this innovation enables the gathering and shortening of the space, time, and cost distance between them [19]. Regarding the impact of urbanization on the diffusion and application of new technologies, Zhang and Huang (2022) found that urbanization can accelerate the diffusion of scientific and technological innovation among large, small, and medium-sized cities and boost the application of new technologies [20]. Therefore, urbanization can promote the production of urban technological innovation and provide an effective diffusion channel for existing technological innovation.

In China, the role of urbanization in technological innovation has inspired interest in the academic community and has been studied from different perspectives. Some research has focused on the impact of urbanization on urban innovation. Cheng (2010) found that urbanization created a suitable environment, accelerated knowledge spillover and innovation diffusion, and thus promoted urban innovation and development [21]. Qiu and Li (2017) examined urban agglomeration in the middle reaches of the Yangtze River in China to analyze the role of urbanization in promoting urban innovation [22]. Liu et al. (2017) focused on innovation efficiency. Their study found that the impact of urbanization on technological innovation efficiency comprises a U-shaped, nonlinear relationship; when the urbanization level crosses a particular critical value, it will promote the improvement of technological innovation efficiency [23]. Another focus of research has been the interaction between urbanization and urban innovation. Cheng and Li (2008) found that China's urbanization and technological innovation exhibit a Granger causality but that urbanization plays a greater role in urban technological innovation. In contrast, urban technological innovation plays a weaker role in urbanization [24]. Zhang et al. (2017) studied the urban agglomeration in China's middle reaches of the Yangtze River. They found a time lag in the dynamic relationship between urbanization and technological innovation. The impact between the two changes from inhibition to promotion over time; further, the impact of urbanization on technological innovation should be stronger than that of technological innovation on urbanization [25]. Ma et al. (2020) studied the Beijing Tianjin Hebei urban agglomeration in China and concluded that the interaction between urbanization and technological innovation is significant. However, they found a large spatial difference in the coupling relationship [26].

Scholars at home and abroad have conducted extensive research on the relationship between urbanization and urban innovation and have made many valuable research achievements. However, there is room for further exploration and discussion based on the existing literature. First, few studies evaluate the impact of China's new urbanization pilot policy on urban innovation. Second, in terms of research methods, most existing studies adopt ordinary panel regression or spatial econometric models. Few researchers regard the comprehensive pilot project of China's new urbanization as a quasi-natural experiment and use the multi-period double difference model (DID) to investigate the policy effect of pilot policies on urban innovation.

Can the pilot policy of new urbanization boost urban innovation? Further data analysis and empirical tests are urgently needed to investigate this research question. Based on the gap in the literature, this paper examines cities at the prefecture level and above from 2008 to 2019 as the research samples. We regard the new urbanization pilot as a quasi-natural experiment and use the multi-period DID model to investigate the policy effect of new urbanization pilot on urban innovation. We provide policy references for comprehensively promoting new urbanization and improving urban innovation capacity. The originality of this paper is as follows: (1) adopting China's new urbanization pilot policy as the research theme; (2) the multi-period DID model is used to analyze the policy effect of new urbanization pilot on urban innovation; (3) our research model tests the intermediary effect of talent gathering, industry gathering, and income scale on urban innovation.

**Point 3:** This study does not theoretically analyze the nexus between the public policy and cities' innovation; therefore, the author should add some contents to improve the theoretical analysis in the revised process.

**3. Answer:** Thanks a lot for your encouragement, as well as your time and very professional and helpful comments. We have added theoretical analysis. Which is shown as below.

**Mechanism analysis**

The modern economy is based on specialization and technological progress. Cities result from economic development, the gathering place of the labor force, capital, and technology. They are the necessary carrier to gathering knowledge and promoting innovation. Cities are thus conducive to generating new knowledge, promoting specialization, and accelerating economic development. This paper expounds the role of the new urbanization pilot on urban innovation from three aspects: agglomeration of human capital, promotion of industrial agglomeration, and expansion of income scale.

(1) **Agglomeration of human capital**

In the urbanization process, many factors at the city level, such as wages, housing prices, employment opportunities, and public services, can impact labor mobility [27]. Generally, the larger a city, the higher the level of its human capital [28]. For example, Bertinelli and Black (2004) studied the relationship between urbanization and human capital promotion by taking 30 years' time series economic data of 100 countries as samples; they found that human capital would increase by 0.144 units on average for every percentage point increase in urbanization, an increase of 0.72 years of schooling on average [29]. China's new urbanization emphasizes "people-oriented" capital. "Population urbanization" is fundamental to quickening the development of high-quality urbanization and promoting the overall development of social harmony and progress. Population gathering provides the advantage of "location proximity" for disseminating and exchanging knowledge, technology, and data [30]. The pilot policy of China's new urbanization identifies specific plans for upgrading vocational skills, including employment skills training, post-skills training, highly skilled talents, and entrepreneurship training, while providing human capital for innovation. The classic endogenous economic growth theory holds that human capital is at the core of technological progress and economic growth; human capital is the core element of innovation output [31]. Therefore, as cities expand, the per capita education level is enhanced, and communication will become more convenient and frequent, which is crucial to improving a city's innovation capacity [32].

(2) **Promoting industrial agglomeration**

Urbanization can provide conditions for industrial economic growth by creating a suitable development environment [33]. Moreover, urbanization and industrial structure upgrades are interrelated; there is a long-term, balanced relationship between urbanization, industrialization, and the development of the tertiary industry [34]. Second, the size and quality of the labor force significantly improves during the urbanization process. High-quality human capital has promoted industrial agglomeration. Urbanization is a process of talent flow, innovative technological production, industrial upgrading. China's new urbanization will change the extensive development mode of traditional urbanization. The key lies in transforming the mode of production, improving the utilization rate of resources, and upgrading and promoting the agglomeration of industrial structures. In the process of industrial agglomeration, many enterprises perform concentrated economic activities in cities, which can lead to local knowledge- and infrastructure-sharing, spillovers, and a larger labor market. This provides the necessary conditions to generate innovation. Therefore, under specific inputs and technology levels, urbanization increases economic efficiency through the industrial agglomeration effect, and promotes a spillover effect to accelerate the diffusion of technological innovation and improve innovation efficiency [35].

(3) **Expanding income scale**

Regarding the impact of urbanization on income, Lewis (1954) put forward the famous "dual economic structure" model, claiming that income inequality distribution will continue to increase during the initial stages of economic development. As urbanization accelerates, the expansion of the urban economy and overall income level will increase, and the shortage of surplus labor will lead to a gradual decline in income inequality [36]. Similarly, the Todaro model (Todaro, 1969) posits that the labor force will gradually transition from rural areas to cities and towns due to the expected income gap between urban and rural areas. The transfer of labor will improve the degree of equalization of labor remuneration, thereby improving the income level of rural residents. Income distribution and its evolution influence the innovation mechanism through demand and subsequently affect the allocation of innovation resources [37]. On the one hand, the widening income gap enables monopoly manufacturers to charge higher product prices from high-income groups, and the price effect strengthens the allocation of innovation resources. On the other hand, income is more concentrated, reducing the proportion of people with purchasing power; then, the market scale effect inhibits R&D and innovation activities [38]. From a dynamic perspective, the revenue growth rate reflects the expected changes in market scale expansion, which accelerates the entry of innovative manufacturers [9] and reduces the uncertainty of expected profits and operational risks, further encouraging R&D innovation [40].

**Point 4:** Policy recommendations in this study are also weak, please improve the contents.

**4. Answer:** Thanks a lot for your very professional and helpful comments. Following the reviewer’s suggestions, we have added discussion on policy recommendations. Which is shown as follows.

This study proposes four policy recommendations based on the above research conclusions: (1) *Summarize effective experience*. Summarize the successful experience regarding the pilot project of new urbanization, especially the effective practices that play a prominent role in improving urban innovation capacity. More emphasis should be placed on the overall improvement of internal quality. Urbanization construction has shifted from increasing quantity and scale to improving low-carbon, science and technology, and other high-quality implications; The government should continue to lead and support increasing urbanization and improving regional innovation capacity. We will promote industrial upgrading, industry city integration, and citizenization of the rural population to create a good foundation for urban innovation.

(2) *Explore multi-dimensional paths*. Exploring the path of new urbanization pilot policies is crucial to promoting urban innovation capability from multiple perspectives and to improve the regional innovation system. First, we suggest improving the flow mechanism of innovative elements and social security mechanisms to promote the agglomeration of high-quality labor. Second, we suggest governments optimize and innovate soft and hard infrastructure, stay current with the development trend of informatization, and improve the regional informatization level. Third, strengthening the public service capacity of the government and non-profit organizations, such as enhancing the rule of law, ensuring fair competition, and protecting intellectual property rights, is key to creating a suitable innovation environment.

(3) *Implement differentiation strategies*. First, improve the urban hierarchy. Key cities should enhance their comprehensive strength and develop their reach and driving capacity. The government should increase support for ordinary cities and provide equal development conditions and opportunities for cities of various levels. Second, promote balanced regional development. The government should encourage cities to strengthen exchanges and collaboration, create opportunities for regional cities to improve their innovation capabilities, and provide dedicated support for cities in the western region. Third, highlight the characteristics of different scales. Large cities should focus on developing modern service and advanced manufacturing industries and enhance the function of scientific and technological innovation. Small and medium-sized cities should develop characteristic industrial clusters, improve infrastructure and public service capabilities, and enhance the attraction of factor agglomeration.

(4) *Develop the intermediary effect*. Research has confirmed that talent gathering, industrial structure, and income scale are the intermediary factors of the new urbanization pilot policy affecting urban innovation. We should develop the intermediary effect of these factors. First, constructing new urbanization should highlight the importance of primary education, health care, social security, and other public services. It should ensure quantity and quality, and improve the overall quality of the urban population. Second, optimizing and upgrading the industrial structure can drive technological innovation. Constructing new urbanization should take this as a new growth point for economic development and promote the integration of urbanization and industrial development. Third, technological innovation requires a large amount of capital investment. Constructing new urbanization should aim to increase the profits of enterprises and personal income to provide financial support for urban innovation.

**Point 5:** Adding limitations and further research directions is also useful.

**5. Answer:** Following the reviewer’s suggestions, we add the limitations of this paper and further research directions.

This study contains some limitations, which can be explored in future research. First, due to data limitations, the urban innovation capability index system constructed in this paper is not comprehensive enough. Only four indicators represent innovation input, and two indicators represent innovation output. The urban innovation capability may be underestimated as a result. Second, based on the data, we cannot further distinguish between specific types of new urbanization, such as population, land, and social urbanization. Distinct types of urbanization may have other impacts on urban creation and could be explored in future research. Third, our preliminary results indicate the short-term effects of the policy under review. In the future, we will further improve the data, measure the urban innovation capacity more accurately, and conduct long-term follow-up surveys regarding the implementation of new urbanization to examine the impact of distinct types of urbanization on urban innovation.

**Point 6:** Language quality of the paper should be improved.

**6. Answer:** Thank you for your valuable questions and professional suggestions. Following the reviewer’s recommendations, we have tried our best to carefully check the expressions. Moreover, the revised manuscript has been professionally proofread by a native English speaker.

We would like to extend our sincere gratitude for these helpful comments.

We greatly appreciate your comments and suggestions that are all valuable and very helpful for revising and improving the quality of our paper.

We have spared no effort to improve the manuscript by making revisions according to the reviewers’ feedback. We have marked the revisions of the paper in the manuscript instead of listing all of them here.

We earnestly appreciate the Editors/Reviewers’ work and hope that our revisions live up to your expectation.

Once again, thank you very much for your comments and suggestions.
