# Supplementary material for: Effect of new urbanization on cities’ innovation in China: Evidence from a quasi-natural experiment of a comprehensive pilot
Source: PLoS One. 2023 Apr 21;18(4):e0284772. doi: 10.1371/journal.pone.0284772 (PMC10121039; doi:10.1371/journal.pone.0284772)
Supplement: Attachment — Submitted filename: review PONE-D-22-18878R1 .docx [file pone.0284772.s002.docx]

Effect of new urbanization on cities’ innovation in China: Evidence from a quasi-natural experiment of a comprehensive pilot

1. In section introduction: You’d better put the National Plan for Innovative Urbanization (2014-2020) at the beginning of the introduction. Then introduce the background of China’ urbanization. Besides, emphasize the importance and significance of Plan.
2. In section mechanism analysis: for each part(human capital; industrial agglomeration; income scale), give the hypothesis at the end paragraph.

Besides, urbanization or Plan is the core explanatory variable, the hypothesis should be introduced about the Plan.

3. For the parallel trend test results, in year of 2019 didn’t pass the test. This need some explanation.
4. This paper analyze three paths, which should correspond to the empirical test. Human capital corresponds to human capital level(hum)? industrial agglomeration corresponds to industrial structure(indu)? how about income scale?
