# Supplementary material for: Effect of new urbanization on cities’ innovation in China: Evidence from a quasi-natural experiment of a comprehensive pilot
Source: PLoS One. 2023 Apr 21;18(4):e0284772. doi: 10.1371/journal.pone.0284772 (PMC10121039; doi:10.1371/journal.pone.0284772)
Supplement: Attachment — Submitted filename: Response to Reviewers.docx [file pone.0284772.s003.docx]

Dear Dr. Abdul Majeed and Reviewers,

Thank you for your and the reviewers’ comments on our manuscript PONE-D-22-18878R1 titled *Effect of new urbanization on cities' innovation in China: Evidence from a quasi-natural experiment of a comprehensive pilot.* Those comments were valuable and very helpful for revising and improving our paper besides providing significant guidance for our research. We have carefully studied the comments and made revisions in the hope of the final approval. The revised portions are marked in red in the paper. The main revisions in the paper and our responses to the reviewer’s comments are as follows:

1. In section introduction: You’d better put the National Plan for Innovative Urbanization (2014-2020) at the beginning of the introduction. Then introduce the background of China’ urbanization. Besides, emphasize the importance and significance of Plan.

**Answer:** Following the reviewer’s suggestions, We have rewritten the literature review.

**1. Introduction**

Promoting new urbanization has become an important strategic goal in China's development plans. To grasp the opportunities provided by urbanization, researchers and policymakers need to examine the new characteristics and trends of urbanization development, explore and optimize the development mode of urbanization, and encourage the innovative vitality of urban and rural economies. In 2014, China released the National Plan for Innovative Urbanization (2014-2020) (hereafter referred to as the Plan). The Plan provides strategic guidance and task scheduling for new urbanization and specifies that policy pilots will be conducted in batches in multiple regions across the country. At the end of 2014, several ministries and commissions jointly released the first batch of national comprehensive pilot plans for new urbanization and the list of pilot areas, and carried out the second and third batch of pilot projects in November 2015 and December 2016. Since the reform and opening up, China's urbanization has made remarkable achievements, but it has also accumulated many outstanding contradictions and problems. The old way of low quality and extensive expansion has been broken, and a new way must be taken. However, China is in a critical period of in-depth urbanization development. It is necessary to firmly grasp the huge opportunities contained in urbanization, accurately study and judge the new trends and characteristics of urbanization development, and properly address the risks and challenges faced by urbanization. To this end, China has put forward the concept of new urbanization and issued the Plan to provide guidance and support for the construction of new urbanization. The Plan is the first urbanization plan issued and implemented in China. It defines the development path, main goals and strategic tasks of urbanization in China for a period of time in the future, and plans for institutional and policy innovation in related fields. It is a macro, strategic and basic plan to guide the healthy development of China's urbanization.

Urbanization is accompanied by industrialization, the rise of non-agricultural industry, and the migration of rural populations to cities and towns. Since the Industrial Revolution, urbanization has become one of the leading factors in economic development, and there is a high correlation between urbanization and economic development. Zhen (2013) found that the average logarithmic correlation coefficient between the urbanization rate and GDP per capita of major developed countries worldwide was 0.85, which confirms the relationship between urbanization level and economic development. In contrast, the correlation coefficient of China from 1978 to 2014 was 0.99, indicating that the correlation between China's economic development and urbanization rate is more prominent [1]. Urbanization has increased China's potential for economic development and has become an essential factor in boosting China's economic growth. Since 1978, China's urbanization has developed rapidly with the gradual breakdown of the urban-rural "dual structure" and the liberalization of population mobility control. By the end of 2021, China's urban population accounted for 64.72% of the total population.

China's traditional urbanization path has been dominated by extensive developments in scale, quantity, and speed [2]. Increased urbanization has led to the rampant expansion of medium- and large-sized cities, degradation of water and soil resources, ecological damage, and other problems, which are becoming increasingly common [3]. Furthermore, this traditional urbanization model, i.e., increasing population proportion and urban area expansion, has also caused economic development problems. For example, slow optimization and adjustment of the industrial structure and the uncoordinated development of industrialization and urbanization have led to a widening income gap between urban and rural residents [4]. The "synergy" between industrial structure and urbanization is not apparent, and the "structural slowdown" of economic development in China has been significant [5]. The urban economy relies too heavily on factor-driven and investment-driven development. It pays insufficient attention to improving urban scientific and technological innovation capacity, leading to the inadequate innovation-driven development capacity of cities [6]. However, China's economy has entered a new stage of high-quality development. The economic development mode oriented by quantitative growth urbanization will not be sustainable during this new stage. Various problems that have accumulated during the process of traditional urbanization need to be solved by changing the direction of urbanization to adjust the mode of economic development. Therefore, the new urbanization policy, which differs from traditional urbanization, is considered the most effective way to achieve high-quality urban economic development [7].

Although urbanization is characterized by the influx of labor, capital, land, and other elements to cities and towns; this leads to positive technology spillover and diffusion effects conducive to promoting urban technological innovation [8]. Moreover, the Plan also proposes that "new urbanization should conform to the new trend of scientific and technological progress and industrial reform, give play to the role of urban innovation carrier, rely on the advantages of science and technology, education and human resources, and promote the city to take the path of innovation-driven development," which provides a viable path to promote urban innovation using the new urbanization policy. However, can implementing the pilot policy of new urbanization promote urban innovation? Does this influence have different heterogeneity? How does this influence arise? This study examines the new urbanization pilot policy as a quasi-natural experiment to address these research questions. We use DID, PSM-DID, intermediary effect, and other methods to analyze the policy impact of new urbanization on urban innovation. Our findings indicate a positive impact on improving our urban innovation system, accelerating the development of innovative cities, and promoting the rational formulation of new urbanization policies.

2. In section mechanism analysis: for each part(human capital; industrial agglomeration; income scale), give the hypothesis at the end paragraph. Besides, urbanization or Plan is the core explanatory variable, the hypothesis should be introduced about the Plan.

**Answer:** Following the reviewer’s suggestions, we have added research hypothesis at the end of the third part.

According to the above theoretical analysis, the new urbanization pilot can promote urban innovation by gathering human capital, promoting industrial agglomeration and expanding income scale. At the same time, the above three mechanisms show that the new urbanization pilot is likely to play a positive role in improving the urban innovation capacity. In order to verify the above analysis, two hypotheses to be tested are proposed here.

**Hypothesis 1:** New urbanization pilot can promote urban innovation capability;

**Hypothesis 2:** The promotion effect of new urbanization pilot on the improvement of urban innovation capacity can be achieved through such channels and mechanisms as gathering human capital, promoting industrial agglomeration and expanding income scale.

3. For the parallel trend test results, in year of 2019 didn’t pass the test. This need some explanation.

**Answer:** Following the reviewer’s suggestions, we have already analyzed this problem.

**5.1. DID applicability analysis**

The premise of the DID model's application is that the treatment and control groups had a common trend before the policy implementation. The credibility of the empirical results using the benchmark model is contingent on the effectiveness of the multiplicative difference method. To test whether this condition was met and to validate the feasibility of the empirical results, the trends in the treatment and control groups were investigated by applying a parallel trend test, as in [45]. Therefore, this study developed the evolution trend diagram of the regional innovation capacity of the treatment and control groups (Fig 1). Figure 1 illustrates that the urban innovation capability of the treatment and control groups had the same trend. Specifically, the coefficients of the regression results before 2014 are not significant. This is sufficient to show that before the pilot policy of new urbanization, the changing trend of the treatment and control groups is consistent, and there was no noticeable difference. However, in 2014 and beyond, the innovation capacity of the treatment group was significantly higher than the control group. Although the regression result coefficient in 2019 is not significant, the main reason is that the implementation time of the Plan is 2014-2020, which is approaching the end. After three batches of urban pilot projects, the experience of new urbanization construction has been summarized and promoted. Non pilot cities have also carried out new urbanization construction, and the policy effect for pilot cities has been basically released. In response to the end of the implementation of the Plan and to provide policy support to continue to strengthen the construction of new urbanization, China issued the National New Urbanization Plan (2021-2035). However, after the implementation of the first (2014) pilot project and the third (2016) pilot project, the innovation ability of the treatment group has changed significantly compared with that of the control group. Therefore, this sample meets the requirements of the parallel trend test, indicating that the empirical results reported by the benchmark model are credible.

4. This paper analyze three paths, which should correspond to the empirical test. Human capital corresponds to human capital level(hum)? industrial agglomeration corresponds to industrial structure(indu)? how about income scale?

**Answer:** According to the questions raised by the reviewers, we make the following description of relevant indicators.

The three path indicators and control variable indicators analyzed in this paper all have different definitions and measurement methods, which are described in detail in this paper, and are excerpted as follows.

(1) In the control variable.

① Human capital level (*hum*) is the proportion of college students in total employment.

② Industrial structure (*indu*) is the proportion of secondary industry output value in GDP.

(2) In the indicators of the path.

① Talent agglomeration effect (*talent*) by the proportion of the non-agricultural employment population of the total urban population.

② The location quotients measure the industrial agglomeration effect (*lq*) [49]. Based on the existing practice [50], the specific expression is as follows:

$lq_{it}=(M_{it}/M_{t})/(P_{it}/P_{t})$ （6）

In formula (6), *lq_it_* represents the location entropy of the secondary industry of city *i* in year *t*. The higher the value, the higher the industry concentration degree in the region. *M_it_* is the number of secondary industry employees in the city in year *t*; *M_t_* is the number of secondary industry employees in the country in year *t*; *P_it_* is the number of urban employees in year *t*; *P_t_* is the number of employees in year *t*;

③ The income scale effect (*pgdp*) is measured by per capita GDP.

We would like to extend our sincere gratitude for these helpful comments.

We greatly appreciate your comments and suggestions that are all valuable and very helpful for revising and improving the quality of our paper.

We have spared no effort to improve the manuscript by making revisions according to the reviewers’ feedback. We have marked the revisions of the paper in the manuscript instead of listing all of them here.

We earnestly appreciate the Editors/Reviewers’ work and hope that our revisions live up to your expectation.

Once again, thank you very much for your comments and suggestions.
